# The Presence of Mutations in the K-RAS Gene Does Not Affect Survival after Resection of Pulmonary Metastases from Colorectal Cancer

**DOI:** 10.1155/2014/157586

**Published:** 2014-02-04

**Authors:** Jon Zabaleta, Borja Aguinagalde, José M. Izquierdo, Nerea Bazterargui, Stephany M. Laguna, Maialen Martin-Arruti, Carmen Lobo, José I. Emparanza

**Affiliations:** ^1^Thoracic Surgery Service, Donostia University Hospital, Paseo Dr. Beguiristain s/n, 20014 Donostia, Gipuzkoa, Spain; ^2^Department of Pathology, Donostia University Hospital, Paseo Dr. Beguiristain s/n, 20014 Donostia, Gipuzkoa, Spain; ^3^Department of Clinical Epidemiology, Donostia University Hospital, CASPe and CIBER-ESP, Paseo Dr. Beguiristain s/n, 20014 Donostia, Gipuzkoa, Spain

## Abstract

*Introduction.* Our objective was to identify mutations in the K-RAS gene in cases of pulmonary metastases from colorectal cancer (CRC) and determine whether their presence was a prognostic factor for survival. *Methods.* We included all patients with pulmonary metastases from CRC operated on between 1998 and 2010. K-RAS mutations were investigated by direct sequencing of DNA. Differences in survival were explored with the Kaplan-Meier method log-rank tests and multivariate Cox regression analysis. *Results. *110 surgical interventions were performed on 90 patients. Factors significantly associated with survival were disease-free interval (*P* = 0.002), age (*P* = 0.007), number of metastases (*P* = 0.001), lymph node involvement (*P* = 0.007), size of the metastases (*P* = 0.013), and previous liver metastasis (*P* = 0.003). Searching in 79 patients, K-RAS mutations were found in 30 cases. We did not find statistically significant differences in survival (*P* = 0.913) comparing native and mutated K-RAS. We found a higher rate of lung recurrence (*P* = 0.040) and shorter time to recurrence (*P* = 0.015) in patients with K-RAS mutations. Gly12Asp mutation was associated with higher recurrence (*P* = 0.022) and lower survival (*P* = 0.389). *Conclusions.* The presence of K-RAS mutations in pulmonary metastases does not affect overall survival but is associated with higher rates of pulmonary recurrence.

## 1. Introduction

In general, the development of cancer is the consequence of a gradual accumulation of genetic alterations. These cause a progressive transformation of normal human cells into malignant cells [1]. The RAS family of genes have the highest known rate of mutations in human cancer, and the aberrant activation of the RAS gene due to a mutation leads to an overexpression of Ras proteins, causing changes in the cells that lead to proliferation, invasion, and metastasis [2].

The conversion of Ras of a protooncogene to an oncogene generally occurs as a consequence of a single mutation in the gene. The mutations are found most often in codon 12 of the gene, followed by condon 13 [3]. In the normal human gene, codon 12 has the sequence CGT that codes for the amino acid glycine (Gly). Any change leading to a loss of the Gly residue at codon 12 may change a normal Ras gene into one that is potentially carcinogenic [3]. Similarly, changes in the Gly residue at codon 13 have the same effect [3].

In recent years, researchers have identified over 20 oncogenes, mutations of which contribute to the occurrence of solid tumours in humans [4]. In colorectal carcinoma, the most common mutations are located in the K-RAS, PIK3CA, BRAF, and N-RAS genes [4]. Recently, Tie reported, in the journal Clinical Cancer Research, that patients with colorectal cancer and K-RAS mutations had a greater risk of pulmonary metastasis [4].

As a result of these findings, in recent years, it has become more common to conduct genetic analysis in cases of pulmonary metastases. Several studies have assessed the relationship between mutation status of the K-RAS gene in primary tumours and in metastatic lesions [5, 6]. To date, however, no published studies have explored mutation status as prognostic factor for survival after metastasectomy. Hence, the purpose of our study was to search for mutations in the K-RAS gene of patients with pulmonary metastases from colorectal cancer and to determine whether the presence of the mutations found was an independent prognostic factor for survival after resection for pulmonary metastases.

## 2. Materials and Methods

### 2.1. Cases

Data from all the patients who underwent surgery for pulmonary metastases of colorectal cancer origin from January 1998 to December 2010 were included in the study. The inclusion criteria were a previous potentially curative resection of colorectal adenocarcinoma (M0 stage and R0 resection of the UICC) and histological confirmation of pulmonary metastasis after thoracic surgery performed with intent to cure (tumour-free resection margins). Cases that represented diagnostic surgery or for which it was not possible to rule out the existence of a primary lung tumour were excluded from the analysis. The follow-up period ended in September 2012, after outpatient clinic visit review or telephone interview with each of the patients. The study was approved by the local Clinical Research Ethics Committee (Ref. no. 02/2012).

Surgical specimens were collected from all patients undergoing resections in this period. Having been fixed in formalin, embedded in paraffin blocks, and stored in the Pathology Department of Donostia University Hospital, the specimens were reviewed by a pathologist to confirm the diagnosis and select a tumour sample for each case.

### 2.2. Surgical Intervention

We followed international criteria to select patients on whom to perform lung metastasectomy: the primary tumour is controlled or is controllable, no extrapulmonary tumour exists, no better method of proven treatment value is available, adequate medical status for the planned resection exists, and complete resection is possible, based on computed tomographic evaluation. Until 2007, we performed thoracotomy and entire lung palpation. Since then, we have treated all unique peripheral colorectal metastases by video-assisted thoracic surgery (VATS). Then, we proceed to make a wedge resection and intraoperative anatomic pathology analysis. If metastatic tissue is identified, we finish the surgery. If the pathologic report cannot confirm metastasis or primary pulmonary neoplasm, we perform a lobectomy. If the lung metastasis is not peripheral, we perform a lobectomy or pneumonectomy. In the intraoperative view, we open the mediastinal pleura and perform lymphadenectomy if we find enlarged lymph nodes. In the preoperative study that continued until 2006, all patients underwent CT scan. Since then, we have routinely performed CT scan and PET-CT.

### 2.3. Extraction of Genomic DNA and K-RAS Sequencing

DNA was extracted from 5 *μ*m thick slices of each tumour sample of the lung, fixed in formalin and embedded in paraffin with the QIAamp DNA FFPE Tissue Kit (Qiagen), in accordance with the manufacturer's instructions.

K-RAS was amplified by PCR with AAA AGG TAC TGG TGG AGT ATT TGA and TGA AAA TGG TCA GAG AAA CC as forward and reverse primers, respectively. The PCR mixture contained 100 ng of DNA from each sample in a total volume of 25 *μ*L containing 0.1 *μ*M of each primer (Bonsai Technology), 0.2 *μ*M of dNTP (Bioline), 1.5 mM of MgCl_2_, 1X buffer, and 0.5 U of Taq DNA polymerase (Kapa Biosystems). The cycle conditions were 2 min at 95°C, followed by 35 cycles of 30 s at 95°C, 30 s at 60°C, and 1 min at 72°C, with a final extension of 2 min at 72°C. To control for contamination, a sample without DNA was included in each PCR. After electrophoresis in 2% agarose gel stained with SYBR Safe (Live Technologies), the amplified products were visualised under ultraviolet light using the Gel Doc XR imaging system (Bio-Rad).

PCR products were sequenced by the service of the Biodonostia Institute Genomics Platform (San Sebastian, Spain) with an ABI PRISM 3130 Genetic Analyzer and analysed with SeqScape software (version 2.5).

### 2.4. Statistical Analysis

A database set up for this study was used to record the following variables: general demographic data, first surgery (site, histological findings, and date), previous resected liver metastases (disease-free interval, histological findings, and resection performed), pulmonary metastasectomy (disease-free interval, surgical access, resection performed, number and size of the metastases resected, lymph node status, complications, and 30-day mortality after surgery), and followup (relapse, reiterative pulmonary metastasectomy, survival, and disease-free survival). For all patients operated on from January 1, 2000, onwards, the levels of CEA prior to thoracotomy were also recorded. Quantitative variables were described using rank, median and mean, and qualitative variables in terms of absolute and relative frequencies as percentages. Survival was analysed by the Kaplan-Meier method with log-rank tests and by multivariate Cox regression analysis using the variables that had a significance of <0.25 in the univariate analysis. The statistical analysis was performed using the SYSTAT 13 package.

## 3. Results

A total of 110 surgical interventions were performed on 90 patients during the study period. The mean age of patients was 65.7 years (median: 66, range: 40–82) and 64 were men.

The mean followup of patients was 58.6 months (median: 52, range: 0–160). During the study period, we did not have any losses to followup, but 49 patients died and there was recurrence in 52 cases. Metastasis was found in the lung in 40 patients, this being the most common metastatic site.

Table 1 reports the main descriptive statistics for the variables concerning characteristics of the patients who underwent surgery and of the surgical interventions performed.

### 3.1. Complications and Mortality

Postoperative complications were observed in eight patients: three cases of intestinal obstruction with ileus and vomiting; two of atelectasis requiring fibrobronchoscopy; one of pneumothorax that resolved after chest drainage; one of empyema that also required chest drainage; and one of haemothorax, the patient being referred for further surgery. Two patients died 30 days after surgery. In both cases, the deaths followed the development of intestinal obstruction with vomiting and aspiration that led to respiratory failure.

### 3.2. Association of Clinical Variables with Overall and Disease-Free Survival

The estimated overall survival (OS) was 84.0 months (95% CI 70.4–97.6) and the estimated disease-free survival (DFS) 64.3 months (95% CI 51.6–76.9), while the mean time to recurrence was 22.9 months (95% CI 15.2–30.6) and 16 patients (17.8%) required lung surgery for the resection of new metastases.

Table 2 lists the variables explored for associations with OS and DFS using univariate analysis and the corresponding levels of significance. After the multivariate analysis (Table 3), the factors found to affect OS were disease-free interval, age, presence of more than one lung metastasis, lymph node involvement, size of the metastases, and previous liver metastasis.

### 3.3. Analysis of K-RAS Mutation Status and Its Relationship with Survival

Of the 90 patients analysed, the DNA obtained was not of sufficiently high quality for sequencing in 11 cases and hence the presence of mutations could not be assessed. Among the others (79 cases), 30 patients were found to have mutations (mutated K-RAS), while in 49 patients no mutations were detected (native K-RAS). The most common mutation was Gly12Asp, found in 12 patients, followed by Gly13Asp and Gly12Val mutations, detected in 7 patients each; a Gly12Ser mutation in 2 patients; and Gly12Cys and Gly12Ala mutations in one patient each.

**Figure 1:**
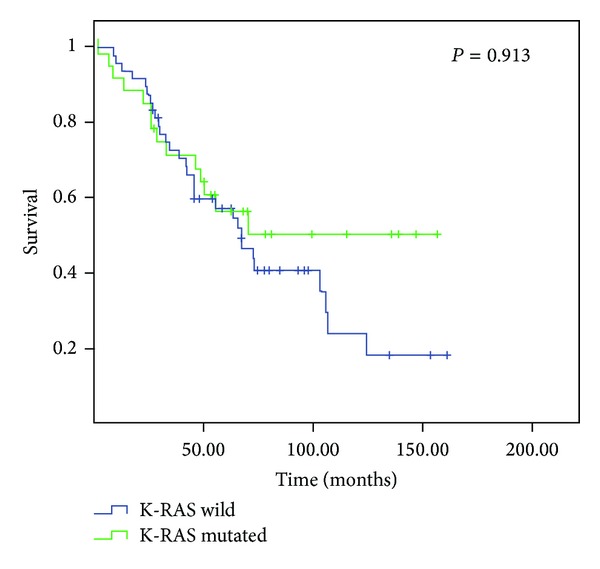
Kaplan-Meier survival curves depending on K-RAS status.

Table 4 shows the main descriptive statistics as a function of K-RAS mutation status. There was no statistically significant difference in OS between the groups with native K-RAS (76.3 months, 95% CI 60.3–92.3) and mutated K-RAS (92.4 months, 95% CI 68.3–116.6) (*P* = 0.913) (Figure 1). Similarly, no statistically significant differences in DFS were detected between these groups (native K-RAS: 59.4 months, 95% CI 44.3–74.4, versus mutated K-RAS: 67.2 months, 95% CI 43.2–91.3; *P* = 0.502).

On the other hand, among individuals with recurrence (52 patients), we found a higher rate of pulmonary recurrence in patients with K-RAS mutations than those with native K-RAS (93.8% versus 66.7%, *P* = 0.040). As for the mean time to recurrence, this was significantly shorter in patients with K-RAS mutations than those with the native gene (16.0 months, 95% CI 4.2–27.1, versus 28.4 months, 95% CI 95 16.7–40.1; *P* = 0.015).

Analysing subgroups with different mutations, the most common type, Gly12Asp, was associated with the highest rate of recurrence of the disease, there being recurrence in 9 of the 12 patients with this mutation. Among those with Gly12Asp, the OS was 47.6 months (95% CI 35.1–59.9), markedly shorter than the mean of 109.7 months when this mutation was absent (95% CI 79.2–140.1, *P* = 0.389). Further, the mean DFS for patients with the Gly12Asp mutation was just 32.6 months (95% CI 17.0–48.3) compared to 86.4 months (95% CI 53.9–118.9) for patients with other mutations (*P* = 0.316). The rate of recurrence in patients with the Gly12Asp mutation was 75.0% (all of them in the lung), and in patients with other mutations the rates of recurrence were 38.9% in any location and 33.3% for lung recurrence, the differences being statistically significant (*P*  = 0.052 and *P*  = 0.025, resp.). Patients in whom the mutation consisted of a substitution of glycine for aspartic acid had a shorter time to recurrence than patients with other mutations: 18.6 months (95% CI 5.2–32.1) compared to 4.7 months (95% CI 2.9–6.4) (*P* = 0.004).

Among the 16 patients who required further surgery, their mutation status was consistently found to remain the same in all cases. On the one hand, eight patients were negative for K-RAS mutations (native genes) at the first and subsequent metastasectomies (two patients requiring two further operations). On the other hand, the other eight patients were found to have K-RAS mutations at the first surgical intervention, the same mutations being detected again at the second and third operations (six and two patients, resp.).

## 4. Discussion

Our results from the survival analysis of 110 consecutive surgical interventions on patients with metastasis from colorectal cancer are partially comparable to those recently published by other authors [7–9]. In particular, our findings suggest that a short disease-free interval and the presence of multiple metastatic lesions are negative predictors of survival, in agreement with the recent reviews of Pfannschmidt et al. [7], Gonzalez et al. [8], and Salah et al. [9]. The meta-analysis published by Gonzalez and colleagues [8] in 2012, including almost 3000 patients who underwent surgery for colorectal metastasis, also indicated that lymph node involvement had an independent impact on survival (as in our series). In addition, the reviews of both Gonzalez et al. [8] and Salah et al. [9] found that elevated levels of carcinoembryonic antigen prethoracotomy were a predictor of a poor prognosis. In our series, although no statistically significant relationship was found in the multivariate analysis, this factor was associated with survival in the univariate analysis.

There still is no consensus on the importance of a history of liver metastasis: in recent years, some publications have indicated that this factor is associated with a significant decrease in survival [10], but other authors did not find such an association [11]. The review of Gonzalez et al. included seven studies that investigated this variable and found a hazard ratio of 1.22 (95% CI 0.91–1.64) [8] for patients with previous liver metastasectomy. These seven studies, however, had quite different characteristics. We believe a multicentre study should be conducted specifically focusing on whether or not a history of metastasis is independently associated with survival.

In most studies, age is used as a descriptive variable and is not often considered as a potential prognostic factor in surgery for pulmonary metastasis. In previous studies, on survival of patients with lung metastases from any origin [12] and those from colorectal cancer [13], we observed a trend towards age being an independent factor that should be taken into account, and the findings of the present study support this idea.

### 4.1. K-RAS Mutation Status

In order to inhibit the activity of the epidermal growth factor receptor (EGFR), anti-EGFR has been included in the therapeutic arsenal for patients with tumours of epithelial origin (lung and colorectal cancer) [14]. The finding that some patients are resistant to such treatments [14], however, has led to an extensive analysis of the signalling cascade associated with EGFR: RAS/RAF/MAPK and to the study of the potential mutations in the RAS gene [15].

Recently, Ross published a review exploring how and when to best conduct K-RAS mutation testing [16]. To optimise the cost-benefit ratio, he recommends not testing all patients with colorectal carcinoma at the outset, but rather performing the analysis in patients with a high risk of recurrence according to the American Joint Committee on Cancer (AJCC) staging system: T2, Stage III, Stage II with transmural invasion, or the presence of metastasis [16].

In our series, 38% of patients had K-RAS gene mutations, a similar rate to those found in other series [4, 5, 17]. It should be highlighted that, to date, the scientific literature documents an association between mutation status and the development of pulmonary metastasis [18, 19], in contrast to liver metastasis that does not seem to be associated with this type of mutation [4, 18, 19]. In our series, we found that patients with mutated K-RAS had a higher rate of pulmonary recurrence than patients with native K-RAS (93.8% versus 66.7%) and that the time to recurrence was shorter in patients with mutated K-RAS.

In recent years, as well as studying the association between K-RAS and pulmonary metastasis, several researchers have investigated the concordance between the mutation status of the primary tumour and related metastatic sites [5, 18]. Cejas and colleagues [18] found that the status was different in 6% of patients: in 71% of these cases the discordance arose from the appearance of mutations in metastatic lesions that had not been found in the primary tumour. In other patients, in whom the primary tumour and the metastasis had different mutations, it has been found that there were different mutations in different regions in the primary tumour [5, 18]. Kobunai and colleagues [5] found complete agreement in mutation status between repeat metastasectomies, as in our study. With these findings, changes in mutation status between primary tumours and metastases in 6–11% of cases [18, 20], and complete agreement in mutation status between repeat metastasectomies [5], the question remains open as to whether it is better to analyse the mutation in the primary tumour or in metastases [5, 18, 20].

So far, K-RAS mutations have been associated with a lack of response to anti-EGFR treatments [14], and though it has not been demonstrated that they reduce OS [18, 21] they do seem to shorten DFS [18]. In our series, we did not find that the presence of mutations had a significant negative influence on overall or disease-free survival.

Considering the various mutations in codons 12 and 13, the most common mutation in our series was Gly12Asp, in agreement with reports to date from studies focusing on primary tumour mutations [5, 18, 22]. In an analysis of the impact of different mutations, Andreyev and colleagues [23] found that the Gly12Val mutation was associated with shorter OS and DFS than the rest of the mutations [23]. In line with this, in 2012, Modest and colleagues [24] concluded that it is essential to classify every mutation individually, given that they observed differences in the survival of patients with mutations in codon 12 or codon 13. In our series, analysing various mutations detected, we found that patients with the Gly12Asp mutation had lower OS and DFS (although these differences were not statistically different) and also a significantly higher rate of recurrence than those with other mutations.

## 5. Conclusions

To conclude, we can state that the presence of a mutated K-RAS gene is not a factor that determines the survival of patients with pulmonary metastases from colorectal cancer. Patients with mutations in this gene do, however, have a higher risk of pulmonary recurrence than those with the native gene. Therefore, analysis of the presence of these mutations in pulmonary metastases may help predict the course of the disease after metastasectomy.

## Figures and Tables

**Table 1:** Characteristics of the 90 patients included.

Variable	Number (percentage)
Origin	
Rectum	56 (62.2%)
Colon	34 (37.8%)
Age	
<65 years	38 (42.2%)
>65 years	52 (57.8%)
Median: 65.7 (range: 40–82), DT: 8.7	
Associated comorbidity	
Absent	38 (42.2%)
Present	52 (57.8%)
Preoperative level of carcinoembryonic antigen	
Negative	55 (61.1%)
Positive	24 (26.7%)
Not measured	11 (12.2%)
Previous liver metastasis	
No	73 (81.1%)
Yes	17 (18.9%)
Disease-free interval	
<12 months	12 (13.3%)
>12 months	78 (86.7%)
Mean: 39.9 (range: 0–181.6), SD: 29.7	
Access	
VATS	7 (6.4%)^a^
Thoracotomy	103 (93.6%)^a^
Lymph node involvement	
No	98 (89.1%)^a^
Yes	12 (10.9%)^a^
Size	
<1 cm	13 (11.8%)^a^
>1 cm	97 (88.2%)^a^
Number of metastases	
1	88 (80.0%)^a^
>1	22 (20.0%)^a^
Laterality of the metastases	
Right	60 (54.5%)^a^
Left	50 (45.5%)^a^
Type of resection	
Atypical	55 (50.0%)^a^
Lobectomy	39 (35.5%)^a^
Lobectomy + atypical	6 (5.5%)^a^
Bilobectomy	1 (0.9%)^a^
Pneumonectomy	5 (4.5%)^a^
Lobectomy with chest wall resection	4 (3.6%)^a^

^a^As a percentage of the 110 surgical interventions performed. VATS: video-assisted thoracic surgery.

**Table 2:** Univariate association of overall and disease-free survival with other variables studied.

Variables	Survival (95% CI)	*P *	DFS (95% CI)	*P *
Previous liver metastasis				
No	89.2 (74.1–104.2)	0.064	70.2 (55.9–84.5)	0.017
Yes	58.8 (34.4–83.1)		35.9 (17.5–54.3)	
DFI				
<12 m	63.6 (31.6–75.6)	0.078	39.4 (17.6–61.2)	0.185
>12 m	89.1 (74.3–103.9)		68.5 (54.5–82.5)	
<24 m	62.5 (47.8–77.1)	0.450	45.5 (31.1–59.8)	0.509
>24 m	89.4 (73.3–105.5)		69.3 (59.8–84.6)	
<36 m	73.1 (57.3–80.9)	0.347	53.0 (37.8–68.2)	0.119
>36 m	95.3 (74.8–115.7)		77.5 (57.9–97.2)	
<48 m	79.8 (63.6–95.9)	0.128	58.8 (43.6–74.1)	0.066
>48 m	99.1 (75.8–122.4)		81.8 (59.2–104.5)	
Sex				
Man	82.5 (67.5–97.6)	0.512	66.2 (50.9–81.5)	0.634
Woman	90.5 (63.9–117.1)		62.7 (39.5–85.9)	
Access				
VATS	84.4 (37.8–130.9)	0.880	83.1 (37.1–128.9)	0.685
Others	84.2 (70.2–98.2)		63.4 (50.4–76.4)	
Complications				
No	87.5 (73.2–101.7)	0.406	68.6 (55.1–82.1)	0.023
Yes	46.9 (32.1–61.8)		27.1 (7.9–46.3)	
Postoperative chemotherapy				
No	51.9 (36.6–67.2)	0.233	33.1 (19.2–46.9)	0.213
Yes	97.6 (81.3–113.9)		73.7 (58.2–89.3)	
Lymph node involvement				
−	85.8 (72.1–99.6)	0.008	67.5 (54.1–80.9)	0.001
+	61.4 (19.8–102.9)		38.6 (1.2–75.4)	
Size				
<1 cm	103.3 (83.4–123.3)	0.057	76.2 (48.8–103.7)	0.198
>1 cm	80.0 (66.2–93.8)		62.2 (48.9–75.4)	
Number				
1	95.1 (79.8–110.4)	0.001	74.9 (59.9–89.8)	0.000
>1	45.9 (29.5–62.4)		27.3 (16.1–38.5)	
Age				
<65 years	104.3 (83.1–125.5)	0.053	72.4 (51.9–92.8)	0.527
>65 years	70.9 (54.9–86.9)		59.6 (43.7–75.4)	
Comorbidity				
No	83.7 (61.6–105.8)	0.033	53.4 (35.1–71.6)	0.040
Yes	87.058 (71.8–102.4)		73.7 (56.7–90.7)	
Laterality of the metastases				
Right	71.4 (59.2–89.6)	0.113	54.2 (39.8–68.7)	0.050
Left	99.5 (75.2–123.3)		84.0 (61.4–106.7)	
Primary				
Rectum	78.3 (62.0–94.6)	0.174	60.3 (44.5–76.0)	0.319
Colon	91.9 (70.7–113.2)		71.8 (51.1–92.4)	
Preoperative level of CEA				
−	99.8 (82.4–117.4)	0.024	75.4 (58.2–92.7)	0.020
+	62.8 (41.1–84.6)		45.9 (25.1–66.9)	

^a^In all cases, time is measured in months. DFI: disease-free interval; VATS: video-assisted thoracic surgery; CEA: carcinoembryonic antigen.

**Table 3:** Multivariate analysis of overall survival.

	Beta coefficient	Type II error	Level of significance	Hazard ratio
Disease-free interval	−0.022	0.007	0.002	0.979 (0.966–0.992)
Age: >65 years	0.977	0.341	0.004	2.657 (1.361–5.184)
Number of metastases	0.673	0.195	0.001	1.960 (1.337–2.874)
Lymph node involvement	1.262	0.468	0.007	3.534 (1.414–8.836)
Size	0.269	0.108	0.013	1.309 (1.059–1.617)
Previous liver metastasis	1.149	0.380	0.003	3.155 (1.497–6.649)

**Table 4:** Association between K-RAS mutation status and the other variables studied.

Variable	Native K-RAS (49 patients)	Mutated K-RAS (30 patients)	*P *
Origin			
Rectum	32 (65.3%)	16 (53.3%)	0.290
Age			
>65 years	31 (63.3%)	16 (53.3%)	0.383
Comorbidity			
Present	25 (51.0%)	20 (66.7%)	0.173
CEA			
Positive	12 (24.5%)	12 (40.0%)	0.245
Previous liver metastasis			
Yes	8 (16.3%)	5 (16.7%)	0.968
Disease-free survival			
>12 months	43 (87.8%)	25 (83.3%)	0.582
Access			
VATS	4 (6.6%)^a^	1 (2.7%)^b^	0.392
Lymph node involvement			
Yes	7 (11.7%)^a^	6 (16.2%)^b^	0.688
Size			
>1 cm	55 (90.2%)^a^	33 (89.2%)^b^	0.528
Number of metastases			
>1	11 (18.0%)^a^	8 (21.6%)^b^	0.520
Laterality			
Right	37 (60.6%)^a^	23 (62.2%)^b^	0.626
Resection			
Atypical	35 (57.4%)^a^	15 (40.5%)^b^	0.622

^a^Of 61 interventions; ^b^of 37 interventions; CEA: carcinoembryonic antigen; VATS: video-assisted thoracic surgery.

## References

[1] Linardou H, Briasoulis E, Dahabreh IJ (2011). All about KRAS for clinical oncology practice: gene profile, clinical implications and laboratory recommendations for somatic mutational testing in colorectal cancer. *Cancer Treatment Reviews*.

[2] Giehl K (2005). Oncogenic Ras in tumour progression and metastasis. *Biological Chemistry*.

[3] Vakiani E, Solit DB (2011). KRAS and BRAF: drug targets and predictive biomarkers. *Journal of Pathology*.

[4] Tie J, Lipton L, Desai J (2011). KRAS mutation is associated with lung metastasis in patients with curatively resected colorectal cancer. *Clinical Cancer Research*.

[5] Kobunai T, Yamamoto Y, Matsuda K (2011). Heterogeneity of KRAS status may explain the subset of discordant KRAS status between primary and metastatic colorectal cancer. *Diseases of the Colon and Rectum*.

[6] Knijn N, Mekenkamp LJM, Klomp M (2011). KRAS mutation analysis: a comparison between primary tumours and matched liver metastases in 305 colorectal cancer patients. *British Journal of Cancer*.

[7] Pfannschmidt J, Dienemann H, Hoffmann H (2007). Surgical resection of pulmonary metastases from colorectal cancer: a systematic review of published series. *Annals of Thoracic Surgery*.

[8] Gonzalez M, Robert JH, Halkic N (2012). Survival after lung metastasectomy in colorectal cancer patients with previously resected liver metastases. *World Journal of Surgery*.

[9] Salah S, Watanabe K, Welter S (2012). Colorectal cancer pulmonary oligometastases: pooled analysis and construction of a clinical lung metastasectomy prognostic model. *Annals of Oncology*.

[10] Landes U, Robert J, Perneger T (2010). Predicting survival after pulmonary metastasectomy for colorectal cancer: previous liver metastases matter. *BMC Surgery*.

[11] Riquet M, Foucault C, Cazes A (2010). Pulmonary resection for metastases of colorectal adenocarcinoma. *Annals of Thoracic Surgery*.

[12] Zabaleta J, Aguinagalde B, Fuentes M, Izquierdo JM, Hernández C, Emparanza JI (2011). Revisión y actualización de los factores pronósticos de las metástasis pulmonares. *Cirugía Española*.

[13] Zabaleta J, Aguinagalde B, Fuentes MG (2011). Survival after lung metastasectomy for colorectal cancer: importance of previous liver metastasis as a prognostic factor. *European Journal of Surgical Oncology*.

[14] Linardou H, Dahabreh IJ, Kanaloupiti D (2008). Assessment of somatic k-RAS mutations as a mechanism associated with resistance to EGFR-targeted agents: a systematic review and meta-analysis of studies in advanced non-small-cell lung cancer and metastatic colorectal cancer. *The Lancet Oncology*.

[15] Ward Y, Wang W, Woodhouse E, Linnoila I, Liotta L, Kelly K (2001). Signal pathways which promote invasion and metastasis: critical and distinct contributions of extracellular signal-regulated kinase and Ral-specific guanine exchange factor pathways. *Molecular and Cellular Biology*.

[16] Ross JS (2012). Clinical implementation of KRAS testing in metastatic colorectal carcinoma: the pathologist’s perspective. *Archives of Pathology & Laboratory Medicine*.

[17] Vaughn CP, Zobell SD, Furtado LV, Baker CL, Samowitz WS (2011). Frequency of KRAS, BRAF, and NRAS mutations in colorectal cancer. *Genes Chromosomes and Cancer*.

[18] Cejas P, López-Gómez M, Aguayo C (2009). KRAS mutations in primary colorectal cancer tumors and related metastases: a potential role in prediction of lung metastasis. *PLoS ONE*.

[19] Sharma N, Saifo M, Tamaskar IR, Bhuvaneswari R, Mashtare T, Fakih M (2010). KRAS status and clinical outcome in metastatic colorectal cancer patients treated with first-line FOLFOX chemotherapy. *Journal of Gastrointestinal Oncology*.

[20] Lamy A, Blanchard F, Le Pessot F (2011). Metastatic colorectal cancer KRAS genotyping in routine practice: results and pitfalls. *Modern Pathology*.

[21] Moosmann N, von Weikersthal LF, Vehling-Kaiser U (2011). Cetuximab plus capecitabine and irinotecan compared with cetuximab plus capecitabine and oxaliplatin as first-line treatment for patients with metastatic colorectal cancer: AIO KRK-0104—a randomized trial of the German AIO CRC study group. *Journal of Clinical Oncology*.

[22] Stinchcombe TE, Der CJ (2011). Are all KRAS mutations created equal?. *The Lancet Oncology*.

[23] Andreyev HJ, Norman AR, Cunningham D (2001). Kirsten ras mutations in patients with colorectal cancer: the “RASCAL II” study. *British Journal of Cancer*.

[24] Modest DP, Jung A, Moosmann N (2012). The influence of KRAS and BRAF mutations on the efficacy of cetuximab-based first-line therapy of metastatic colorectal cancer: an analysis of the AIO KRK-0104-trial. *International Journal of Cancer*.

